# Phytoremediation Potential of *Crotalaria pumila* (Fabaceae) in Soils Polluted with Heavy Metals: Evidence from Field and Controlled Experiments

**DOI:** 10.3390/plants13141947

**Published:** 2024-07-16

**Authors:** Miguel Santoyo-Martínez, Patricia Mussali-Galante, Isela Hernández-Plata, Leticia Valencia-Cuevas, Alexis Rodríguez, María Luisa Castrejón-Godínez, Efraín Tovar-Sánchez

**Affiliations:** 1 Doctorado en Ciencias Naturales, Universidad Autónoma del Estado de Morelos, Av. Universidad No. 1001, Col. Chamilpa, Cuernavaca 62209, Morelos, Mexico; miguel.santoyo425@gmail.com; 2 Laboratorio de Investigaciones Ambientales, Centro de Investigación en Biotecnología, Universidad Autónoma del Estado de Morelos, Av. Universidad No. 1001, Col. Chamilpa, Cuernavaca 62209, Morelos, Mexico; alexis.rodriguez@uaem.mx; 3 Facultad de Ciencias Biológicas, Universidad Autónoma del Estado de Morelos, Av. Universidad No. 1001, Col. Chamilpa, Cuernavaca 62209, Morelos, Mexico; hallowyn31@yahoo.com.mx (I.H.-P.); mlcastrejon@uaem.mx (M.L.C.-G.); 4 Escuela de Estudios Superiores del Jicarero, Universidad Autónoma del Estado de Morelos, Carretera Galeana-Tequesquitengo s/n, Comunidad El Jicarero, Jojutla 62915, Morelos, Mexico; leti70477@yahoo.com.mx; 5 Centro de Investigación en Biodiversidad y Conservación, Universidad Autónoma del Estado de Morelos, Av. Universidad No. 1001, Col. Chamilpa, Cuernavaca 62209, Morelos, Mexico

**Keywords:** bioaccumulation, phytoextraction, phytostabilization, herbaceous plants, mining tailings, heavy metals, morphological effects

## Abstract

Phytoremediation is a useful, low-cost, and environmentally friendly alternative for the rehabilitation of heavy-metal-contaminated (HM) soils. This technology takes advantage of the ability of certain plant species to accumulate HMs in their tissues. *Crotalaria pumila* is a herbaceous plant with a wide geographical distribution that grows naturally in environments polluted with HMs. In this work, the bioaccumulation capacity of roots and leaves in relation to five HMs (Cr, Cu, Fe, Pb, and Zn) was evaluated, as well as the morphological changes presented in *C. pumila* growing in control substrate (without HMs) and mine-tailing substrate (with HMs) under greenhouse conditions for 150 days. Four metals with the following concentration pattern were detected in both tissues and substrates: Fe > Pb > Cu > Zn. Fe, Pb, and Zn concentrations were significantly higher in the roots and leaves of individuals growing on mine-tailing substrate compared to the control substrate. In contrast, Cu concentration increased over time in the exposed individuals. The bioconcentration factor showed a similar pattern in root and leaf: Cu > Fe > Pb > Zn. Around 87.5% of the morphological characters evaluated in this species decreased significantly in individuals exposed to HMs. The bioconcentration factor shows that *C. pumila* is efficient at absorbing Cu, Fe, and Pb from the mine-tailing substrate, in the root and leaf tissue, and the translocation factor shows its efficiency in translocating Cu from the roots to the leaves. Therefore, *C. pumila* may be considered as a HM accumulator plant with potential for phytoremediation of polluted soils with Cu, Pb, and Fe, along with the ability to establish itself naturally in contaminated environments, without affecting its germination rates. Also, it exhibits wide geographical distribution, it has a short life cycle, exhibits rapid growth, and can retain the mine-tailing substrate, extracting HMs in a short time.

## 1. Introduction

Mining is a primary economic activity that generates waste, called mine tailings, that can contain heavy metals (HMs). These are commonly considered as those elements that have a specific density of more than >5 g/cm^3^ [1], such as cadmium (Cd), copper (Cu), chromium (Cr), iron (Fe), lead (Pb), and zinc (Zn). HMs may be bioavailable in soils; this facilitates the absorption over time in the exposed organisms (bioaccumulation), which may facilitate the access and bioaccumulation of HMs along the food chain (biomagnification) [2,3,4,5,6]. Hence, plants are the primary access route for HMs into biological systems [4,7]. In plants, some HMs produce toxic effects in high concentrations, such as Ni and Cu. However, other HMs are highly toxic at lower concentrations, such as Cd, Cr, Pb, Al, and Hg [8,9]. Overall, exposure to HMs can cause various adverse effects such as a delay in seed germination and in root growth, a reduction in aerial biomass (stem and leaf), micromorphological alterations such as changes in the stomatal and trichome density, as well as alterations in biochemical processes. All these adverse effects may result in poor seedling development [10,11,12,13,14,15,16].

The bioaccumulation of HMs in plants varies among different plant organs, populations, plant species, and taxonomic groups [8,9,17,18]. Moreover, bioaccumulation is determined by the metal retention capacity, the plant–root–metal interaction, the plant metabolism, the metal bioavailability, and the soil physicochemical properties, including pH, electrical conductivity, and the organic matter content [19,20,21,22].

Despite the harmful effects of HMs on plants, some species have efficient detoxification processes. These plants avoid toxic HM effects through the translocation of metals to the aerial part and their storage in cell walls and organelles such as vacuoles, as well as in structures such as the epidermis, trichomes, and even the cuticle, where HMs cause less damage to the photosynthesis process [23,24,25]. Hence, many species tolerate high metal concentrations because they restrict their root absorption and translocation to the leaves, allowing them to maintain relatively low concentrations in the aerial biomass, regardless of the metal concentration in the soil (exclusion strategy). On the other hand, other plant species actively absorb metals from the soil and accumulate them in non-toxic forms in their aerial biomass (accumulative strategy) [26,27,28]. In this context, different degrees of bioaccumulation have been recognized, ranging from those that can only do so in low concentrations to those that can resist exceptionally high concentrations of HMs without showing any visible signs of toxicity [26,27,29,30,31].

In the phytoremediation processes of soils polluted by HMs, the use of local species is recommended for the preservation of genetic integrity and the conservation of regional diversity, and mainly in mine tailings where there are physical, chemical, and biological limitations to the establishment of plants. Therefore, the search for native HM accumulating plants, capable of storing HMs in different structures such as root, stem, and leaf, and able to be implemented in phytoremediation strategies for polluted environments, is essential. In this sense, it has been documented that the bioconcentration factor (BCF), which measures the efficiency of a plant species in accumulating metals from the soil in its tissues, and the translocation factor (TF), which indicates the efficiency of the plant in transporting the metal from the root to its aerial tissues [32], are useful indicators for selecting species with phytoremediation potential. For example, TF and BCF have been used to propose some plant species as heavy metal accumulators, such as *Phyla nodiflora* (L.) Greene (Verbenaceae), *Rubus fruticosus* L. (Rosaceae), *Sesbania herbacea* (Mill.) McVaugh (Fabaceae), *Gentiana pennelliana* Fernald (Gentianaceae) [33], and *Polygonum thunbergii* Siebold and Zucc. (Polygonaceae) [34]. On the other hand, it has also been suggested that plant species with both a BCF and a TF greater than one (both) have the potential to be used in phytoextraction. Moreover, plants with a BCF greater than one and a TF less than one (BCF > 1 and TF < 1) have the potential for phytostabilization [33]. In general, there is a continuous interest in searching for native plant species that bioaccumulate HMs in their tissues along with tolerating their adverse effects [35,36].

Huautla, Morelos, Mexico has been an area known for its mining activity since the sixteenth century, and through until 1988. During the 18th and 19th centuries, around six mines were exploited intermittently in the region. In the 1950s, the company “Exploradora de Minas S.A.” exploited four mines in the area. Later, between 1976 and 1988, the company “Rosario de México, S.A.” exploited other mines, obtaining mainly silver and lead. All these mines are currently not in operation, and Huautla is located within the Sierra de Huautla Biosphere Reserve (REBIOSH is the acronym in Spanish). As a result of those activities, it is estimated that there are approximately 780,000 tons of waste in the zone, forming mine tailings rich in Pb, Cd, As, Zn, Cu, Fe, and Cr. These residues were left in the open air without any treatment [37,38].

Bioavailable metals have been reported in these residues, which have contaminated the natural resources of the region, having adverse effects on the health of the human population [39]. Additionally, adverse effects have been documented in arthropod and microarthropod diversity [40,41], as well as our noting a reduction in the population density of small mammals [38], and alterations in the morphological, physiological, and genetic characters of plant species [36]. Therefore, it is essential to identify new plant species that have remediation abilities, primarily, local species that naturally establish in this polluted zone.

*Crotalaria pumila* Ortega (Fabaceae) is a herbaceous species that establishes naturally in environments contaminated with HMs. *C. pumila* has high germination rates, rapid growth, is a dominant plant in the herbaceous stratum, and has a wide geographical distribution in Mexico (from temperate zones to arid and semi-arid zones). These attributes may be considered for plant selection in phytoremediation strategies [9]. However, to date, it is unknown if this species is capable of bioaccumulating heavy metals when it grows on polluted soils, and the probable effects on macro- and micromorphological characters. Moreover, this species has a primary use as food, and its cultivation is promoted for self-consumption [42,43]. Therefore, if this species bioaccumulates HMs, it may pose a potential risk if introduced into the food chain and into human dietary food.

The aim of the present work was to analyze if *C. pumila* bioaccumulates heavy metals in different tissues in order to know if this plant species may be an appropriate candidate for phytoremediation strategies in soils polluted with HMs. Therefore, this study evaluated: (a) seed germination percentage of *C. pumila* individuals from exposed and control sites; (b) the bioaccumulation of Cr, Cu, Fe, Pb, and Zn in root and leaf tissues of individuals growing on mine-tailing substrate and control substrate in greenhouse conditions; (c) the effect of bioaccumulation of HMs on macro- and micromorphological characters of *C. pumila* individuals growing on mine-tailing substrate and on control substrate in greenhouse conditions; and (d) to analyze the potential of *C. pumila* for phytoremediation of HM-polluted soils.

## 2. Results

### 2.1. Germination of Crotalaria pumila Seeds from Control and from Sites Exposed to Heavy Metals

The germination percentages showed that the seed origin (control vs. exposed) does not influence the germination percentage (*F*_1,20_ = 1.09, *p* = 0.30). However, pregerminative treatment has a significant effect on germination (*F*_1,20_ = 525.64, *p* < 0.0001). Likewise, there was no significant effect based on the site per-treatment interaction (*F*_1,20_ = 0.19, *p* = 0.66; Table 1). The results show that the seeds from both sites (control and exposed) have less than 10% germination when they do not receive a pregerminative treatment. In contrast, when the seeds from both sites undergo mechanical scarification, their germination percentage is higher than 90% (Table 1).

### 2.2. Bioaccumulation of Heavy Metals in the Root and Leaf Tissue of Crotalaria pumila

Of the five HMs analyzed (Cr, Cu, Fe, Pb, and Zn), the presence of Cu, Fe, Pb, and Zn was only detected in the root and leaf tissue of *C. pumila* individuals, while Cr was not detected. Two-way analysis of variance showed a significant effect of exposure time (t) on Zn concentration in leaf tissue. Meanwhile, the treatment (T) showed significant differences on Fe, Pb, and Zn concentrations in root and leaf tissues, but, in the case of Cu, it only showed significant differences in the root. The interaction (t × T) showed significant differences in the Zn concentration in roots and in leaves and for Pb concentration in roots (Table 2).

The bioaccumulation values observed in *C. pumila* individuals are shown in Table 2 (both experimental groups showed a difference between their life cycles). The individuals growing in the control substrate concluded their life cycle in 90 days, while the individuals growing in the mine-tailing substrate finished their life cycle 60 days later, on day 150. Regarding bioaccumulation, it was found that Cu levels were significantly higher in the root and leaf tissue of the individuals growing in the control substrate compared to individuals growing in the mine tailings. It remained constant over time, except for the concentration in the roots in exposed individuals; on day 150, an increase was shown (Table 2).

Concerning Fe concentration between treatments, individuals growing on mine-tailing substrate presented a higher bioaccumulation in the analyzed tissues (root and leaf). Additionally, Fe concentration in both evaluated structures of control and exposed individuals did not change throughout the exposure time. Notably, Fe concentration in the leaves of exposed individuals decreased on day 120 (Table 2).

Lead concentrations presented significant differences between treatments, observing the highest levels in the root and leaf tissue of exposed individuals with respect to the control individuals, the latter without showing changes through the exposure time. While the Pb concentration in the root and leaf tissue of the exposed individuals increased their levels through the exposure time, on day 150, the highest concentration was found in both structures (Table 2).

In the case of Zn, the concentration values between treatments denoted significant differences in both structures, presenting the highest levels in the exposed individuals. However, the root and leaf concentration of exposed individuals decreased over the exposure time, while the root concentration of control individuals increased over time. The leaf concentration of control individuals was oscillatory, and the highest concentration occurred on day 60 (Table 2).

### 2.3. Bioconcentration Factor (BCF) and Translocation Factor (TF) of Heavy Metals in Roots and in Leaves from Crotalaria pumila Individuals Growing on Mine-Tailing Substrate

To estimate the BCF and TF values (Table 3) we used the root and leaf metal concentrations reported in Table 2. The BCF presented the following pattern: Cu > Fe > Pb in root and leaf tissue in *C. pumila* individuals growing on mine-tailing substrate. The values for Fe and Pb in the roots were higher in comparison to those found in the leaves. In contrast, the BC of Cu in the leaf tissue was more than that in the roots (Table 3), and the Zn values were reduced in both evaluated structures. On the other hand, the TF presented the following pattern: Cu > Pb > Fe > Zn, where Cu showed an average value of 1.2, while the Pb, Fe, and Zn values were lower than 1.0 (Table 3).

### 2.4. Macro- and Micromorphological Changes in Crotalaria pumila Individuals Growing under Greenhouse Conditions in the Control and Mine-Tailing Substrate over Time

The analysis indicated that of the 16 morphological characters evaluated, 87.5% denoted significant differences between treatments (T) in individuals of *C. pumila* growing under greenhouse conditions in two treatments (control and mine-tailing substrate). In general, all characters from plants growing on the tailing substrate showed a reduction. Only the petiole diameter (DP) and 1/3 apical width (AW) did not show significant effects from the treatment (T) (Table 4).

On the other hand, the *C. pumila* individuals grown in mine-tailing substrate showed a significant increase over time (t) in 50% of the morphological characters evaluated (Table 4). Individuals growing on control substrate showed significant differences over time (t) in only 37.5% of the morphological characters evaluated. The stomatal index (SI) was higher in *C. pumila* individuals growing on the control substrate in comparison with individuals growing on the mine-tailing substrate (Table 4).

## 3. Discussion

### 3.1. Bioaccumulation of Heavy Metals in C. pumila

*Crotalaria pumila* individuals bioaccumulated four of the five HMs analyzed (Cu, Fe, Pb, and Zn) in root tissue and in leaves, out of which only Pb is a non-essential element. In the present study, the Cu bioaccumulation in exposed individuals showed small differences over time, presenting root bioaccumulation values of 74 to 92 mg kg^−1^, while, in the case of the leaves, there were no significant differences over time in the Cu bioaccumulation, which showed a mean of 93.5 mg kg^−1^. Copper is an essential metal for adequate growth and development in plants. However, copper excess induces the release of reactive oxygen species and free radicals, leading to DNA damage, protein degradation, photosynthesis efficiency decline, and cell death. In response to the stress caused by Cu bioaccumulation, different proteins and molecules are induced to cope with the Cu toxicity. For example, phytochelatins and proline are produced at the root level to immobilize Cu excess and reduce its translocation to aerial tissues [44,45,46]. While, at the leaf level, different metallothioneins are expressed; some of them are implicated in Cu detoxification [15,47,48], and these proteins may be involved in the maintenance of a stable concentration of Cu in the leaf tissue of *C. pumila*.

Overall, this study shows that *C. pumila* (Fabaceae) can accumulate Cu in the leaf tissue (86.6 to 98.4 mg kg^−1^) at similar concentrations to the Cu bioaccumulation that has been reported in plant species with a different life form than the herbaceous form, for example, in arboreal species *Gliricidia sepium* (Jacq.) Kunth ex Walp (Fabaceae) and *Dodonaea viscosa* L. Jacq. (Sapindaceae) the level is about 94.2 mg kg^−1^ and 84.9 mg kg^−1^, respectively. These species are included in families that have been reported as accumulators and hyperaccumulators of heavy metals [18,49]. Also, Cu bioaccumulation has been reported in other plant species with a herbaceous life form. For example, in leaves of *Alectra sessiliflora* (Vahl) O. Ktze. (Orobanchaceae), Cu, 45 to 769 mg kg^−1^ [50]; *Elsholtzia haichowensis* Sun. (Lamiaceae), Cu, 17 to 391 mg kg^−1^ [51]; and *Haumaniastrum robertii* (Robyns) P.A. Duvign. and Plancke (Lamiaceae), Cu, 62 to 6159 mg kg^−1^ [52].

Due to the Cu bioaccumulation differences between the diverse life forms, it is important to characterize or find new species for phytoremediation of contaminated sites. Currently, phytoremediation studies suggest the inclusion of different life forms for a better recovery of those sites [9].

The bioaccumulation of Fe in root and leaf tissues was higher in individuals growing in the exposed substrate. Furthermore, the Fe concentration in the leaves of exposed individuals decreased over time. However, when comparing structures, a greater bioaccumulation was observed in the root than in the leaf of the exposed individuals. Swapna and colleagues [53] reported a similar pattern in *Chromolaena odorata* (L.) R.M. King and H. Rob. (Asteraceae), finding that the higher bioaccumulation of this metal was observed in the root (750 mg kg^−1^) compared to the leaf (80 mg kg^−1^).

Iron is an essential metal for plant development and physiology due to its crucial role in the photosynthesis process, as well as in the biosynthesis of hemeproteins and essential biomolecules such as chlorophyll [54,55]. However, Fe high-level bioaccumulation may cause adverse effects related to reactive oxygen species release [56,57,58]. Copper bioaccumulation in the root is a tolerance mechanism to prevent its excessive bioaccumulation in the aerial part of the plants [59]. Different plants, such as *Centella asiatica* (L.) Urb. (Apiaceae) (Bhat et al. 2016), *Jatropha curcas* L. (Euphorbiaceae) [60], *Pelargonium hortorum* L.H. Bailey (Geraniaceae) [61], *Typha latifolia* L. (Typhaceae) [62], and *Sanvitalia procumbens* Lam. (Asteraceae) [63], can cope with Fe toxicity via bioaccumulation in the roots.

In the case of Zn and Pb, a higher concentration was observed in exposed individuals of *C. pumila* in both evaluated structures. For Zn, exposed individuals decreased their concentration over time for both structures, while for Pb, an increase in its concentration was detected in both structures of the exposed individuals through the exposure time. Lead and zinc showed a higher root mean concentration in exposed individuals, 365.8 and 247.7 mg kg^−1^, respectively, in comparison with the concentration determined in the leaf tissue, 272.2 and 86.4 mg kg^−1^, respectively. Similar results were reported by Mussali-Galante and colleagues [18] for Zn, who described a similar bioaccumulation process in *Gliricidia sepium* (root 67.1 mg kg^−1^ and leaf 0.6 mg kg^−1^).

Zinc is an essential micronutrient for plant growth and development. For example, Zn is necessary for photosynthesis, membrane integrity, phytohormones, and nucleic acids synthesis, as well as being a cofactor required for the structure and function of numerous enzymatic proteins [64,65]. The Zn concentrations are variable in the different aerial structures according to the developmental stage of the plant. Zinc is absorbed through the root system and then is translocated to the aerial part of the plants. Zn is generally stored in the stem tissues, and later mobilized to the growing tissues. In plants, Zn is redistributed from senescing tissues to young shoots, reproductive organs, and seeds; the seeds are considered reservoirs of this metal [66,67]. It has been reported that several plant species can accumulate high Zn levels in their roots, rather than in their leaves and shoots, as a mechanism to avoid the adverse effects of Zn overload. Also, it has been reported that Zn bioaccumulation in plants is differential between flowering and post-flowering stages; additionally, old leaves show lower Zn concentrations in contrast to young leaves [68].

Concerning Pb, it has been reported that the root structure is where the highest Pb concentration is accumulated. Xiong [69] reported that *Sonchus oleraceus* L. (Asteraceae) accumulates higher Pb concentrations in the root (1113.2 mg kg^−1^) compared to those in the leaf (65.7 mg kg^−1^). For their part, Rotkittikhun and colleagues [70] documented higher Pb concentrations in the root in comparison to the metal concentrations in the leaves in 26 plant species such as Asteraceae, Cyperaceae, Euphorbiaceae, Fabaceae, Malvaceae, and Poaceae. These findings could be explained by the fact that Pb is absorbed by the roots and retained by the cell walls of the root cells. Further, Pb translocation to the aerial part of the plant is limited due to the absence of specific transporters. In general, only 3% of the Pb in the root is translocated to the leaf, with minimal concentration increase over time, because Pb is a non-trace element [19]. It has also been documented that Cr and Pb affect the cell cycle, which induces the inhibition of cell division, causing alterations to root growth, which could have consequences in shoot development, affecting the normal development of the plant [71].

The observed bioaccumulation pattern in *C. pumila* individuals revealed that Fe, Pb, and Zn bioaccumulated mainly in the roots, showing a limited translocation to the aerial tissues. In plants, the root system is responsible for the uptake of heavy metals from the soil. In the root environment, chelating molecules, transport proteins, and plant induced soil pH changes mediate the metal uptake process [72]. Therefore, the root system plays a crucial role in limiting the distribution of heavy metals into the aerial parts for avoiding the toxic effects of excessive bioaccumulation in the shoot system [73]. Lastly, it should be mentioned that the presence of HMs in control individuals of *C. pumila* growing in the region’s substrate is related to the fact that the municipality of Tlaquiltenango presents a natural richness of sulfur minerals in soil (mainly silver and lead). Commonly found minerals are arsenopyrite (FeAsS), galena (PbS), acanthite (Ag2S), and calclacite (Cu2S) [37,74,75].

### 3.2. Effects of Heavy Metals on the Root, Stem, and Leaf Morphology of C. pumila

In the present study, 16 macro- and micromorphological characters were evaluated, out of which 87.5% decreased in individuals growing in mine-tailing substrate compared to individuals growing in the control substrate. Similar results were reported in species exposed to HMs with a herbaceous life form. Tovar-Sánchez and colleagues [27] documented, for *Zea mays* L. (Poaceae), a reduction of 50% of the evaluated leaf traits, while Rosas-Ramírez [63] and Sidhu and colleagues documented a decrease in the root size, leaves, and biomass for *S. procumbens* and *Chenopodium murale* L. (Amaranthaceae), respectively.

Lead was one of the metals that bioaccumulated in *C. pumila*, increasing its concentration over time in exposed individuals in both evaluated structures. The root bioaccumulated the highest Pb concentration, a fact that accounts for the observed effects on the morphological traits evaluated in the exposed individuals. Similar results were reported by Rosas-Ramírez [63] in the herbaceous *S. procumbens*, where the bioaccumulation of Pb in roots was 980 mg kg^−1^ while in leaves it was 760 mg kg^−1^, with morphological characters such as root length, stem, and biomass being affected. Similar results have been reported in other studies of plants exposed to Pb [*S. oleraceus* [69] and *Acalypha indica* L. (Euphorbiaceae)] [76].

The results of the stomatal index in individuals of *C. pumila* growing in mine-tailing substrate differed significantly from those growing in control substrate, presenting a lower index in exposed individuals. These results are similar to those for other species exhibiting shrubby life, such as *Vachellia campechiana* (Mill.) Seigler and Ebinger (Fabaceae) [36] and *Prosopis laevigata* (Humb. and Bonpl. ex Willd.) M.C.Johnst. (Fabaceae), both arboreal life species, where a decrease in the number of stomata per unit area has been documented [35]. This can be explained by a response of the plant to avoid excess gas exchange, increasing its stomatal resistance [77,78].

In the present study, the exposed individuals showed decreases in the root length, stem size, and biomass, as well as in the macro- and micromorphological characters of the leaves (Table 4). The observed effects may be related to the bioaccumulation of Cu, Fe, Pb, and Zn in *C. pumila*. Our results coincide with those reported for *Z. mays* (Poaceae) plants growing in the same study site as the present work [39]. Despite the bioaccumulation of heavy metals in these species, both settle, grow, and develop in this polluted zone.

The effects on morphological characters could be attributed not only to metal but to the interaction between metals. Mainly, studies under controlled conditions have shown that exposure to heavy metals, individually or as mixtures (the type of metal or concentrations), differentially affects plants, either by modifying the concentrations of other essential or non-essential metals, the biochemical processes, or the morphological characters, since metals in combination may show antagonistic or synergistic interactions [79,80,81]. Due to the mixes of heavy metals present in the various contaminated sites, it is crucial to expand the options of plants that can be used to remediate those sites.

### 3.3. The Use of C. pumila for Bioremediation of Copper-Contaminated Soils

It has been reported that for phytoremediation studies, herbaceous species are one of the most evaluated plants [9,82] due to their rapid growth, large amount of biomass, strong resistance, and effective stabilization of soils [83]. Also, they are pioneer plants and usually are adapted to unfavorable conditions such as low soil-nutrient content, stressful environments, and shallow soils [51,84,85]. However, in most of the studies that have evaluated the bioaccumulation of HMs in plants, species belonging to the Brasicaceae family have been selected, a herbaceous group that inhabits temperate and cold zones.

The present study focused on understanding the ability of *C. pumila*, a herbaceous species belonging to the Fabaceae family with an annual life cycle, to accumulate heavy metals [86]. Species of the Fabaceae family have characteristics that make them resilient to environmental stress conditions, like those present in heavy-metal polluted environments. Different Fabaceae have been proposed for revegetation and phytostabilization of mine soils since they can produce high biomass, have fast growth rates, and can accumulate different HMs in their tissues [87]. *Crotalaria pumila* has a broadly natural distribution in America [88], in Mexico, and in the Huautla region. In addition, it has been reported as a pioneer species in heavy-metal impacted sites [89] that are home to diverse types of vegetation such as xerophilous scrub, tropical deciduous forests, and temperate forests [90]. Different heavy-metal phytoremediation studies propose the introduction of native species because the introduction of foreign species may cause a threat to the ecological dynamics of the impacted site, a factor mainly related to the possibility of an invasive profile of the introduced species [91]. Given the wide distribution of *C. pumila*, its introduction to stabilize contaminated soils could reduce the negative impact on the whole ecosystem. All these attributes, and the results found in this study, suggest that *C. pumila* could be considered as a candidate for phytoremediation strategies in multi-metal-contaminated soils.

Exposure to HMs, such as Ni, Pb, Cu, and Cd, affects several signaling mechanisms in the germination process [14,15]. For example, Cu affects the cotyledonary carbohydrate status in the germination of bean seeds [92]. However, the germination percentages of *C. pumila* seeds were not affected by their site of origin; the seeds from both control and exposed sites reached values close to 100%. Similar findings have been reported for the bush *V. campechiana* that also inhabits the mining area of Huautla, Morelos [36].

On the other hand, the bioaccumulation of HMs is differentiated between structures, roots, leaves, or the entire plant. It has been considered that plant species with both a BCF and a TF greater than one (both) have the potential to be used in phytoextraction. Moreover, plants with a BCF greater than one and a TF less than one (BCF > 1 and TF < 1) have the potential for phytostabilization [33]. In the case of *C. pumila*, it can potentially be used in strategies for phytoextraction for sites with Cu since it showed values for BCFroot, BCFleaf, and TF greater than one. Meanwhile, *C. pumila* can potentially be used in strategies for phytostabilization for sites with Fe and Pb (Table 4). Other studies have considered that if a plant presents values of TF > 1, as well as BCF > 1, the species is considered an accumulator for the analyzed metal [93,94,95]. In the case of *C. pumila*, the BCF and TF values were >1, so it could be considered as an accumulator plant mainly of Cu. The levels of TF in *C. pumila* are similar to those in other herbaceous species reported to have Cu accumulating potential, such as *Taraxacum officinale* F.H. Wigg. (Asteraceae) (TF = 1.2 Cu), *Phyla nodiflora* (L.) Greene (Verbenaceae) (TF Cu = 12.0, TF Zn = 1.1), *R. fruticosus* (TF Cu = 5.6), and *Sesbania herbacea* (Mill.) McVaugh (Fabaceae) (TF Cu = 4.0) [33,96].

## 4. Materials and Methods

### 4.1. Crotalaria pumila Seed Collection Sites

**Figure 1:**
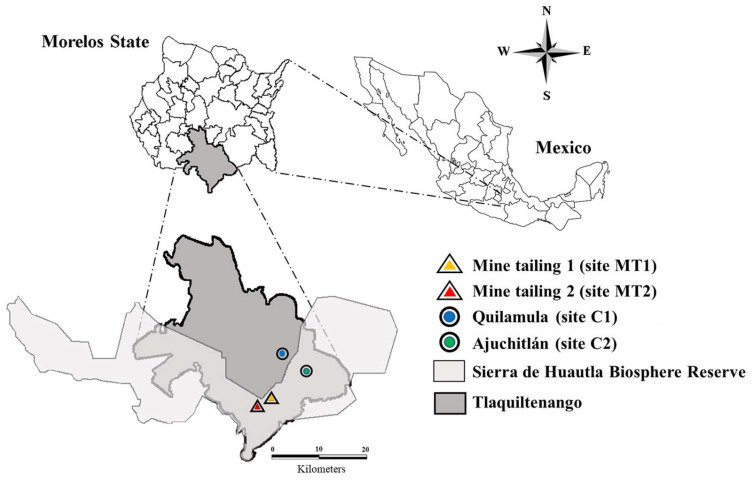
Geographical distribution of the two study sites at the Sierra de Huautla Biosphere Reserve, Morelos, Mexico. Control site (triangle) and exposed site (circle).

The seed collection was carried out in the town of Huautla, municipality of Tlaquiltenango, Morelos. In this town, mining activity was focused on the exploitation of Pb, Zn, and Ag, forming three tailings rich in heavy metals. The exposed sites selected were mine tailing 1 (site MT1), which is located 500 m from Huautla town and is the largest in the area (18°26′36.37″ N–99°01′26.71″ W) and mine tailing 2 (site MT2), which is located 1000 m from the nearest population (18°2′22.62″ N–99°01′51.71″ W, Figure 1). Also, two control sites were chosen: Quilamula (site C1), located at 18°30′52″ N and 98°59′59″ W and Ajuchitlán (site C2), located at 18°27′52″ N and 98°58′53″ W (Figure 1). Both sites have a sub-humid temperate climate with summer rains and average annual precipitation of 900 mm. The yearly average temperature exceeds 22 °C, and the predominant vegetation type is deciduous tropical forest. The forest is characterized by presenting a marked seasonality where the trees do not exceed 15 m in height and lose their leaves in the dry season [90]. The control sites are more than six kilometers away in a straight line from the exposed sites; they present ecological and geographical characteristics similar to the exposed sites and do not show a record of mining activity or metallic contamination due to anthropogenic activity. Moreover, the water streams and predominant winds flow in a north–south direction [38]. Hence, neither the winds nor the watercourses can be dispersing the pollutants to the control sites. The HM concentrations in the control substrate were Cu (1.99 mg kg^−1^), Fe (117.27 mg kg^−1^), Pb (3.27 mg kg^−1^), and Zn (0.24 mg kg^−1^), while the concentrations in the tailing substrate were Cu (8 mg kg^−1^), Fe (80 mg kg^−1^), Pb (46 mg kg^−1^), and Zn (428 mg kg^−1^) [35,36].

### 4.2. Study Species

**Figure 2:**
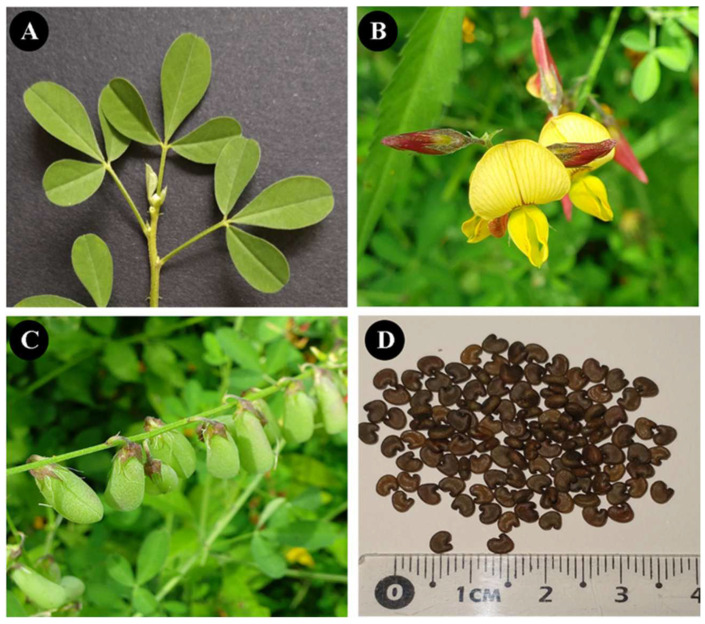
Principal structures of *Crotalaria pumila*. (**A**) Leaflet, (**B**) flowers, (**C**) fruits, and (**D**) seeds.

*Crotalaria pumila* is an annual herbaceous species of the Fabaceae family. It grows upright or upward, with stems up to 50 cm long. It presents trifoliate leaves; leaflets are 3.2–5.0 cm long, linear, elliptical or obovate, cuneate or rounded base, and with sub-rounded apex. Its phenology of flowering and fruiting occurs from May to December. The flowers are arranged in clusters and the fruit is an inflated legume 15 mm long by 8 mm in diameter with asymmetric kidney-shaped seeds 1.5–2.6 mm long and 1.7–2.8 mm wide, greenish-brown, yellowish-green, or coffee colored (Figure 2) [97]. It is a species native to Mesoamerica, which is distributed from the southern United States of America to South America. In Mexico, it is widely distributed, both in deciduous tropical forests and also in temperate forests and xerophilous scrubs [43]. *Crotalaria pumila* is a plant species that survives, grows, and reproduces naturally in soils polluted with HMs. Also, this species grows in small, aggregated groups of plants, an attribute that reduces soil erosion in the substrates where it is established, and which can be useful to limit the dispersion of HMs contained in the mine tailings. Finally, it is a species that has a primary use as food, and its cultivation is promoted for self-consumption [42,43].

### 4.3. Seed Collection and Germination

Because HM exposure can alter the germination process, resulting in low fitness of plant individuals, it is an important factor that needs to be taken into account when considering plants for phytoremediation or phytoextraction strategies. *Crotalaria pumila* seeds were collected from individuals established at the study sites (control and exposed) following the protocol reported by Gold and colleagues [98]. At the study sites, 20 individuals were randomly chosen, from which 20% of the ripe fruits were collected. Mature fruits were transported to the laboratory; the seeds were extracted, cleaned, and selected, eliminating those parasitized by insects. We collected seeds from both control (C1 and C2) and exposed sites (MT1 and MT2), then, the collected seeds per treatment (control vs. exposed) were homogenized.

To evaluate the germination percentages of *C. pumila*, the seeds—from the control and the exposed sites—were subjected to mechanical scarification due to their physical latency. Twenty-five seeds were sown in Petri dishes using 1% agar-agar as substrate using six replicates per treatment, evaluating this test over 20 days.

After seed germination, 72 seedlings were placed in individual polyethylene bags for nurseries (5 L capacity) with treatment substrates: 36 for the mine-tailing substrate and 36 for the control substrate. Soil from Quilamula was used as the control substrate; it was sieved with a stainless-steel sieve with a mesh aperture of 0.2 mm (Fiicsa brand, Mexico City, Mexico) to obtain a substrate texture similar to the mine tailings. As a mine-tailing substrate, a mixture of mine tailings from the exposed sites (MT1 and MT2) was used. The plants were kept under greenhouse conditions at a temperature that ranged from 32 to 35 °C and were watered twice a day, three times a week. The obtained plants were used to evaluate the bioaccumulation of HMs and to measure macro- and micromorphological characters.

### 4.4. Evaluation of Macro- and Micromorphological Characters

Six individuals were randomly selected per each treatment (control and mine-tailing substrate) to evaluate the effect of HM exposure on the morphological characters of *C. pumila*. Root and stem length and root and leaf biomass (dry and fresh) were measured for each individual. Subsequently, six mature leaves per individual were randomly chosen to evaluate the macro- and micromorphological characters shown in Table 1. These evaluations were carried out after every 30 days of exposure to the treatments until the 150 days period was accomplished.

Foliar macromorphological characters were evaluated with a digital Vernier (stainless hardened) and a digital scale (Acculab Scales, Titusville, NJ, USA). For micromorphological characters, a foliar epidermal impression was made using the cyanoacrylate glue replica technique (Table 5). Per individual, three lamellae were made with epidermal impressions of the abaxial part of the leaf. The lamellae were observed with a 40X light microscope (Leica, Schweiz, CH) with bright-field illumination (BF) and differential interference contrast (DIC). From each slide, three photomicrographs were randomly taken. Finally, from nine microphotographs, the number of stomata and the number of epidermal cells were obtained per individual. With these data, the stomatal index (SI) was determined, which was calculated according to Salisbury [99].

SI = (SN/SN + NEC) × 100

where:

SI = stomatal index
SN = number of stomatal cells per area unit
NEC = number of ordinary epidermal cells per area unit

**Table 5:** Macromorphological characters analyzed in *Crotalaria pumila*.

Abbreviation	Character	Units
Size characters		
RL	Root length	cm
SL	Stem length	cm
FRB	Fresh root biomass	g
DRB	Dry root biomass	g
FLB	Fresh leaf biomass	g
DLB	Dry leaf biomass	g
Macromorphological characters		
LBL	Leaf blade length	mm
WLB	Width of the leaf blade	mm
LP	Length of the petiole	mm
DP	Petiole diameter	mm
LIV	Length of the intermediate vein	mm
WIV	Width of the intermediate vein	mm
1/3AW	1/3 Apical width	mm
1/3BW	1/3 Basal width	mm
CLB	Coverage of leaf blade	mm^2^
Micromorphological characters		
SI	Stomatal index	mm^2^

### 4.5. Heavy-Metal Determination

Ten samples of the mine-tailing substrate were analyzed to determine the level of five metals (Cr, Cu, Fe, Pb, and Zn). The samples were dried and sieved following the methodology of the NMX-AA-132-SCFI-2006 Mexican standard [100]. This process consists of adding 50 mL of 0.01 mol dm^−3^ CaCl_2_ to 10 g of substrate. The sample was left stirring for 24 h and centrifuged at 1500 rpm for 15 min (Eppendorf Centrifuge 5804R, Eppendorf, Hamburg, Germany), followed by recovering of the supernatant by filtration. The metal concentrations in the substrate samples were determined by atomic absorption spectrometry using the flame method (GBC 908 A, GBC Scientific Equipment Pty Ltd., Melbourne, Victoria, Australia).

For metal quantification (Cr, Cu, Fe, Pb, and Zn) in the root and leaf tissue of *C. pumila*, three samples were taken from six individuals per substrate (mine-tailing and control) after every 30 days of exposure. Root samples were thoroughly washed with running tap water and rinsed with distilled water three times to remove any soil particles attached to the root surfaces; to corroborate this, the roots were observed under a stereoscopic microscope. Then, 0.25 g of each structure was pulverized and poured into containers previously washed with HNO3. The samples were subjected to acid digestion using a Microwave Accelerated Reaction System (CEM^®^ MARS-5, CEN, Matthews, NC, USA), using 10 mL of 70% (*v*/*v*) HNO_3_ aqueous solution in closed Teflon bombs. The samples were dissolved and filtered in distilled water to a final volume of 50 mL until analysis was performed. A non-tissue sample was simultaneously processed and used as a negative control. Finally, metal concentrations were analyzed by atomic absorption spectrophotometry using the flame method (GBC 908 A, GBC Scientific Equipment Pty Ltd., Victoria, AU). The concentration of the five analyzed metals was determined by calibration curves obtained using internal standard solutions of pure metal ions (ULTRA SCIENTIFIC INSTRUMENTS, AGILENT, Santa Clara, CA, USA). The standard calibration curves showed correlation coefficients (R^2^) between 0.99 and 1. For plants and substrates, the recovery percentage ranged, for all the elements, between 95.7 and 103%, with RSD values lower than 10%. The minimum detection limits according to the manufacturer for Cr, Cu, Fe, Pb, and Zn were 0.003, 0.001, 0.005, 0.0015, 0.01, and 0.0005 mg/L, respectively. The samples from the exposed and control sites were processed simultaneously by triplicate.

### 4.6. Statistical Analysis

A two-factor analysis of variance was performed [101] to assess the effect of the site (control and exposed), treatment (mechanical scarification and no treatment), and site per-treatment interaction on seed germination of *C. pumila*. In addition, a Tukey test was performed to establish significant differences in the averages between sites and treatments [100].

Likewise, a two-factor analysis of variance was performed to determine the effect of the exposure time (30, 60, and 90 days), treatment (substrate: control and mine-tailing), and time per-treatment interaction on the bioaccumulation of Cu, Fe, Pb, and Zn in root and leaf tissue of *C. pumila* individuals. Subsequently, a Tukey test was carried out to calculate significant differences between pairs of average of morphological characters assessed over time in both treatments [101].

Likewise, two-factor analysis of variance was performed to determine the effect of the exposure time (30, 60, and 90 days), treatment (substrate: control and mine-tailing), and time per-treatment interaction on the variation in 16 morphological characters (15 macro- and one micromorphological). Exposure times of 120 and 150 days were excluded from the two-factor analysis because individuals grown on control substrate finished their life cycle earlier. Subsequently, a Tukey test was carried out to determine significant differences between average pairs of morphological characters assessed over time in both treatments [101]. All analyses were performed using the STATISTICA 8 program (Statsoft Inc, Tulsa, OK, USA) [102].

The HM phytoextraction capacity of *C. pumila* was evaluated using the bioconcentration factor (BCF) and the translocation factor (TF), according to Olguín and Sánchez [32], and Ali and colleagues [28]. These indices were calculated as follows:

BCFroot = Croot/Cmine tailing

BCFleaft = Cleaft/Cmine tailing

TF = Cleaft/Croot

where Cmine tailing is the concentration in the mine tailing, Cleaft is the concentration of the metal detected in the leaf tissue, and Croot is the concentration of the metal detected in the root tissue.

## 5. Conclusions

The results obtained in this work showed that *C. pumila* might be an appropriate candidate for phytoremediation (phytoextraction and phytostabilization) of environments polluted with Cu, Pb, and Fe because this species presents high seed germination percentages and has the ability to bioaccumulate Cu, Fe, and Pb in its tissues. Despite the changes in most of the morphological characters, the survival of the individuals was not diminished and their phenological period was longer than the plants in the control substrate, a fact that is important when considering this species for phytoremediation strategies because it means that it can spend more time growing on the polluted soil, favoring substrate retention and immobilization as well as bioaccumulating more HMs from soils. Other characteristics that strengthen *C. pumila* as a phytoremediator species are its wide geographical distribution, encompassing three types of vegetation (deciduous tropical forests, temperate forests, and xerophilous scrubs); natural establishment in contaminated environments; high recruitment rates; rapid growth; short life cycle; and efficient control of tailings’ erosion. Therefore, it is important to use native plants with the characteristics of *C. pumila* for phytoremediation of heavy-metal polluted soils since they are often efficient in terms of survival, growth, and reproduction in environmental conditions that are under stress.

## Figures and Tables

**Table 1:** Seedling percentage (Mean ± SE) of *Crotalaria pumila* from the control and the exposed sites, under pregerminative treatments.

Site	Treatment	Seedling (%)
Exposed	No scarification	6.00 ± 1.71 a
	Mechanical scarification	97.33 ± 1.23 b
Control	No scarification	4.00 ± 2.07 a
	Mechanical scarification	97.67 ± 1.23 b

Different lower-case letters denote significant differences between treatments (Tukey *p* < 0.05).

**Table 2:** Heavy-metal concentration (Mean ± standard deviation; mg kg^−1^) in roots and leaves of *Crotalaria pumila*, growing in reference substrate and tailing substrate.

	Treatment									
	Root					Leaf				
Time (days)	Control		Exposed		SDT	Control		Exposed		SDT
Cooper (Cu)										
30	99.91 ± 6.52	a	73.95 ± 5.89	A	*	98.34 ± 5.47	a	86.59 ± 3.61	A	**
60	102.60 ± 7.41	a	80.09 ± 2.86	A	**	101.98 ± 4.35	a	96.71 ± 4.70	A	ns
90	104.75 ± 9.02	a	76.17 ± 4.45	A	**	100.79 ± 5.08	a	90.71 ± 4.95	A	*
120	-		82.69 ± 2.98	AB		-		98.43 ± 4.83	A	
150	-		92.51 ± 3.47	B		-		95.19 ± 5.16	A	
ANOVA										
Time (t)			*F*_2,15_ = 0.60 ns			*F*_2,15_ = 0.42 ns				
Treatment (T)			*F*_1,15_ = 71.05 ***			*F*_1,15_ = 1.76 ns				
t × T			*F*_2.15_ = 0.39 ns			*F*_2,15_ = 0.16 ns				
Iron (Fe)										
30	566.61 ± 39.03	a	731.78 ± 19.24	A	***	479.68 ± 17.87	a	665.32 ± 41.44	A	**
60	548.87 ± 21.46	a	795.91 ± 27.69	A	***	428.42 ± 30.91	a	613.41 ± 21.03	A	**
90	576.40 ± 26.97	a	778.08 ± 34.59	A	***	441.25 ± 37.81	a	631.55 ± 30.68	A	ns
120	-		831.51 ± 21.02	A		-		450.93 ± 23.78	B	
150	-		776.89 ± 29.65	A		-		460.76 ± 28.67	B	
ANOVA										
Time (t)			*F*_2,15_ = 1.31 ns			*F*_2,15_ = 0.349 ns				
Treatment (T)			*F*_1,15_ = 265.55 ***			*F*_1,15_ = 11.67 **				
t × T			*F*_2,15_ = 11.19 ns			*F*_2,15_ = 0.02 ns				
Lead (Pb)										
30	241.32 ± 12.85	a	362.09 ± 20.50	A	**	261.20 ± 24.14	a	266.13 ± 15.27	A	ns
60	295.02 ± 19.27	a	312.42 ± 25.59	A	ns	290.52 ± 16.68	a	246.53 ± 17.89	A	ns
90	244.63 ± 18.54	a	353.12 ± 17.65	A	***	288.72 ± 13.03	a	236.09 ± 20.81	A	*
120	-		394.70 ± 18.36	B		-		261.94 ± 18.82	A	
150	-		406.83 ± 26.16	B		-		350.41 ± 15.78	B	
ANOVA										
Time (t)			*F*_2,15_ = 0.54 ns			*F*_2,15_ = 0.08 ns				
Treatment (T)			*F*_1,15_ = 31.20 ***			*F*_1,15_ = 0.15 *				
t × T			*F*_2.15_ = 3.87 *			*F*_2,15_ = 0.04 ns				
Zinc (Zn)										
30	18.31 ± 3.23	a	260.71 ± 17.96	A	***	26.84 ± 4.01	a	214.48 ± 18.05	A	***
60	7.06 ± 2.65	b	278.15 ± 16.48	A	***	36.01 ± 6.06	b	82.16 ± 9.15	B	***
90	36.56 ± 9.05	c	234.77 ± 10.94	AB	***	22.98 ± 5.36	c	56.28 ± 7.56	C	***
120	-		266.22 ± 14.36	A		-		36.73 ± 5.07	D	
150	-		198.71 ± 18.78	B		-		42.28 ± 7.01	CD	
ANOVA										
Time (t)			*F*_2,15_ = 5.93 ns			*F*_2,15_ = 92.45 ***				
Treatment (T)			*F*_1,15_ = 9868.98 ***			*F*_1,15_ = 327.83 ***				
t × T			*F*_2.15_ = 90.85 ***			*F*_2,15_ = 93.04 ***				

Different lower-case letters denote significant differences between control individuals during treatment time; different upper-case letters denote significant differences between exposed individuals during treatment time; * = *p* < 0.05, ** = *p* < 0.01, and *** = *p* < 0.001; ns = not significant; and SDT = statistical differences between treatments.

**Table 3:** Bioconcentration factor (BCF) values and translocation factor (TF) values of Cu, Fe, Pb, and Zn in roots and leaves of *Crotalaria pumila* from individuals growing on tailing substrate during treatment.

	Concentration (mg kg^−1^)			
Time (days)	Tailing	BCF Root	BCF Leaf	TF
Copper (Cu)				
30	8	9.24	10.82	1.17
60	8	10.01	12.09	1.21
90	8	9.52	11.34	1.19
120	8	10.34	12.30	1.19
150	8	11.56	11.90	1.03
mean ± SD		10.1 ± 0.91	13.5 ± 0.60	1.16 ± 0.07
Iron (Fe)				
30	80	9.15	8.32	0.91
60	80	9.95	7.67	0.77
90	80	9.73	7.89	0.81
120	80	10.39	5.64	0.54
150	80	9.71	5.76	0.59
mean ± SD		9.79 ± 0.45	7.05 ± 1.26	0.73 ± 0.15
Lead (Pb)				
30	46	7.87	5.79	0.73
60	46	6.79	5.36	0.79
90	46	7.68	5.13	0.67
120	46	8.58	5.69	0.66
150	46	8.84	7.62	0.86
mean ± SD		7.95 ± 0.81	5.92 ± 0.99	0.74 ± 0.08
Zinc (Zn)				
30	428	0.61	0.50	0.82
60	428	0.65	0.19	0.30
90	428	0.55	0.13	0.24
120	428	0.62	0.09	0.14
150	428	0.46	0.10	0.21
mean ± SD		0.58 ± 0.07	0.20 ± 0.17	0.34 ± 0.27

Note: To estimate BCF and TF values, we used the root and leaf concentration values reported in Table 2.

**Table 4:** Mean ± standard error of macro- and micromorphological characters from *Crotalaria pumila* growing in greenhouse conditions on tailing substrate and reference substrate.

Character	Time (days)	Treatment				SDT	ANOVA	
		Control		Exposed				
Size characters								
Root length								
	30	22.33 ± 2.11	a	8.15 ± 1.16	A	*	Time (t)	*F*_2,30_ = 7.23 ***
	60	23.33 ± 2.76	a	13.22 ± 0.47	B	*	Treatment (T)	*F*_2,30_ = 8.90 **
	90	19.83 ± 2.24	a	13.00 ± 1.06	B	ns	t × T	*F*_2,30_ = 31.46 ***
	120	-		18.24 ± 1.21	C			
	150	-		21.52 ± 2.93	D			
Stem length								
	30	17.83 ± 1.78	a	7.67 ± 0.87	A	*		
	60	26.00 ± 1.15	b	15.73 ± 1.30	B	*	Time (t)	*F*_2,30_ = 7.32 ***
	90	26.00 ± 0.88	b	11.50 ± 0.76	AB	*	Treatment (T)	*F*_1,30_ = 7.80 **
	120	-		20.22 ± 1.11	C		t × T	*F*_2.30_ = 24.51 ***
	150	-		21.67 ± 2.47	C			
Fresh root biomass								
	30	1.61 ± 0.70	a	1.04 ± 0.40	A	ns		
	60	0.35 ± 0.08	a	0.20 ± 0.03	A	ns	Time (t)	*F*_2,30_ = 1.48 ns
	90	0.31 ± 0.05	a	0.40 ± 0.07	A	ns	Treatment (T)	*F*_1,30_ = 3.27 ns
	120	-		0.89 ± 0.12	B		t × T	*F*_2,30_ = 3.17 ns
	150	-		1.42 ± 0.01	C			
Dry root biomass								
	30	0.25 ± 0.06	a	0.10 ± 0.06	A	ns		
	60	0.12 ± 0.06	a	0.07 ± 0.06	AB	*	Time (t)	*F*_2,30_ = 0.28 ns
	90	0.09 ± 0.08	a	0.09 ± 0.06	AB	ns	Treatment (T)	*F*_1,30_ = 3.39 ns
	120	-		0.17 ± 0.03	B		t × T	*F*_2,30_ = 1.96 ns
	150	-		0.30 ± 0.23	C			
Fresh leaf biomass								
	30	7.11 ± 1.35	a	0.30 ± 1.35	A	*		
	60	6.17 ± 1.35	a	1.76 ± 135	B	**	Time (t)	*F*_2,30_ = 0.32 ns
	90	6.51 ± 1.91	a	3.19 ± 1.35	C	*	Treatment (T)	*F*_1,30_ = 16.61 *
	120	-		4.51 ± 0.30	C		t × T	*F*_2,30_ = 0.76 ns
	150	-		4.92 ± 1.31	D			
Dry leaf biomass								
	30	2.00 ± 0.46	a	0.09 ± 0.46	A	ns		
	60	1.93 ± 0.46	a	0.55 ± 0.46	AB	*	Time (t)	*F*_2,30_ = 0.34 ns
	90	1.84 ± 0.66	a	1.12 ± 0.47	B	ns	Treatment (T)	*F*_1,30_ = 10.52 *
	120	-		1.71 ± 0.48	C		t × T	*F*_2,30_ = 0.64 ns
	150	-		1.68 ± 0.49	C			
Macromorphological characters								
Leaf blade length								
	30	19.09 ± 0.60	a	13.45 ± 0.50	A	***		
	60	15.44 ± 0.37	b	13.50 ± 0.51	A	**	Time (t)	*F*_2,210_ = 15.02 ***
	90	16.22 ± 0.47	b	10.87 ± 0.32	B	***	Treatment (T)	*F*_1,210_ = 109.32 ***
	120	-		11.87 ± 0.29	BC		t × T	*F*_2,210_ = 9.34 ***
	150	-		12.90 ± 0.32	C			
Width of the leaf blade								
	30	9.04 ± 0.31	a	6.13 ± 0.22	A	***		
	60	7.22 ± 0.22	b	6.28 ± 0.24	A	**	Time (t)	*F*_2,210_ = 14.59 ***
	90	6.98 ± 0.60	b	4.88 ± 0.29	B	***	Treatment (T)	*F*_1,210_ = 66.34 ***
	120	-		4.87 ± 0.29	B		t × T	*F*_2,210_ = 6.55 **
	150	-		4.88 ± 0.13	B			
Length of the petiole								
	30	15.88 ± 0.61	a	11.04 ± 0.57	A	***		
	60	12.79 ± 0.31	b	11.23 ± 0.60	A	*	Time (t)	*F*_2,210_ = 5.05 **
	90	14.79 ± 0.83	ab	9.22 ± 0.34	B	***	Treatment (T)	*F*_1,210_ = 77.96 ***
	120	-		9.27 ± 0.30	B		t × T	*F*_2,210_ = 7.85 **
	150	-		9.31 ± 0.30	B			
Petiole diameter								
	30	0.53 ± 0.02	a	0.54 ± 0.02	A	ns		
	60	0.41 ± 0.02	b	0.52 ± 0.02	AB	***	Time (t)	*F*_2,210_ = 7.63 ***
	90	0.46 ± 0.02	ab	0.47 ± 0.02	B	ns	Treatment (T)	*F*_1,210_ = 70.75 ***
	120	-		0.47 ± 0.02	B		t × T	*F*_2,210_ = 4.30 *
	150	-		0.46 ± 0.16	B			
Length of the intermediate vein								
	30	12.67 ± 0.42	a	9.66 ± 0.36	AB	***		
	60	9.54 ± 0.33	b	9.76 ± 0.41	A	ns	Time (t)	*F*_2,210_ = 10.28 ***
	90	10.72 ± 0.35	b	8.43 ± 0.42	B	**	Treatment (T)	*F*_1,210_ = 24.98 ***
	120	-		8.39 ± 0.23	B		t × T	*F*_2,210_ = 9.71 ***
	150	-		8.38 ± 0.20	B			
Width of the intermediate vein								
	30	6.66 ± 0.22	a	4.93 ± 0.20	A	***		
	60	5.35 ± 0.22	b	5.00 ± 0.22	A	ns	Time (t)	*F*_2,210_ = 4.02 *
	90	5.52 ± 0.42	ab	4.62 ± 0.39	A	ns	Treatment (T)	*F*_1,210_ = 18.14 ***
	120	-		4.02 ± 0.10	B		t × T	*F*_2,210_ = 3.53 *
	150	-		4.18 ± 0.10	AB			
1/3 Apical width								
	30	18.31 ± 0.97	a	12.74 ± 0.66	A	***		
	60	11.78 ± 0.44	b	16.82 ± 0.60	B	***	Time (t)	*F*_2,210_ = 4.61 *
	90	13.21 ± 0.50	b	13.52 ± 0.54	A	ns	Treatment (T)	*F*_1,210_ = 0.02 ns
	120	-		9.43 ± 0.29	C		t × T	*F*_2,210_ = 33.96 ***
	150	-		9.64 ± 0.29	C			
1/3 Basal width								
	30	16.01 ± 0.79	a	10.46 ± 0.62	A	***		
	60	9.13 ± 0.29	b	10.12 ± 0.43	A	ns	Time (t)	*F*_2,210_ = 31.84 ***
	90	8.37 ± 0.38	b	10.60 ± 0.39	A	***	Treatment (T)	*F*_1,210_ = 2.85 ns
	120	-		5.49 ± 0.18	B		t × T	*F*_2,210_ = 29.23 ***
	150	-		5.99 ± 0.18	B			
Coverage of leaf blade								
	30	217.00 ± 6.91	a	151.06 ± 5.95	A	***		
	60	174.91 ± 3.71	b	153.01 ± 0.96	A	**	Time (t)	*F*_2,210_ = 3.23 ***
	90	187.51 ± 6.71	b	123.26 ± 3.40	B	***	Treatment (T)	*F*_1,210_ = 115.73 ***
	120	-		128.37 ± 2.94	B		t × T	*F*_2,210_ = 10.45 ***
	150	-		126.28 ± 3.08	B			
Micromorphological characters								
Stomatal index (SI)								
	30	25.07 ± 0.42	a	22.50 ± 0.47	A	***	Time (t)	*F*_2,210_ = 10.15 ***
	60	26.01 ± 0.42	ab	23.88 ± 0.32	AB	***	Treatment (T)	*F*_1,210_ = 43.41 ***
	90	27.20 ± 0.59	b	24.54 ± 0.47	B	***	t × T	*F*_2,210_ = 0.21 *
	120	-		24.39 ± 0.35	B			
	150	-		24.33 ± 0.42	B			

Different lower-case letters denote significant differences between control individuals during treatment time (Tukey *p* < 0.05); different upper-case letters denote significant differences between exposed individuals during treatment time (Tukey *p* < 0.05); and SDT = statistical differences between treatments, ns = not significant, * = *p* < 0.05, ** = *p* < 0.01, and *** = *p* < 0.001.

## Data Availability

Please contact author for data request.
